# Delayed Closure of Open Abdomen in Septic Patients Treated With Negative Pressure Vacuum Therapy and Dynamic Sutures: A 10-Years Follow-Up on Long-Term Complications

**DOI:** 10.3389/fsurg.2020.611905

**Published:** 2021-01-15

**Authors:** Anna Theresa Hofmann, Christopher May, Karl Glaser, René H. Fortelny

**Affiliations:** ^1^Department of General, Visceral and Oncological Surgery, Klinik Ottakring, Vienna, Austria; ^2^Medical Faculty, Sigmund Freud Private University, Vienna, Austria

**Keywords:** open abdomen, negative pressure wound therapy, delayed fascial closure, dynamic fascial suture, incisional hernia

## Abstract

**Introduction:** Patients with open abdomen after surgical interventions associated with the complication of secondary peritonitis are successfully treated with negative pressure wound therapy. The use of dynamic fascial sutures reduces fascial lateralization and increases successful delayed fascial closure after open abdomen treatment.

**Methods:** In 2017 we published the follow-up results of 38 survivors out of 87 open abdomen patients treated with negative pressure wound therapy and dynamic fascial sutures between 2007 and 2012. In our current study we present the 10-years follow-up results regarding long-term complications with the focus on incisional hernias and pain. Since 2017 seven more patients have died, hence 31 patients were included in the current study. The patients were asked to answer questions about specific long-term complications of OA treatment including pain, the presence of incisional hernias and subsequent surgical interventions. Demographic data and data regarding fascial closure after open abdomen treatment were collected. All results were analyzed quantitatively. The follow-up period was 8–13 years.

**Results:** The median age was 69 (30–90) years, and 15 (48.4%) were females. Twenty-four patients (77.4%) responded to the questionnaire: Three patients (12.5%) suffered from pain in the original operating field, all three at rest but not during exercise. None of the patients required analgesic treatment. Eleven patients (45.8%) were found to have incisional hernias. Five out of 11 hernias (45.5%) were treated by surgery and did not declare any pain in the operating field. Among the patients with incisional hernias lower MPI (Mannheimer Peritonitis Index) at the time of primary surgery but more reoperations and treatment days were found. The technique of fascial closure was heterogenic and no differences in the occurrence of incisional hernia could be detected.

**Conclusion:** The incidence of incisional hernias after open abdomen treatment is still high, but are associated with little pain in the original operating field. Further studies are required to investigate methods for fascial closure techniques after OA treatment.

## Introduction

The treatment of patients with open abdomen (OA) and the subsequent fascial closure (FC) are still challenging clinical problems. The mortality rate in patients with abdominal sepsis remains between 20 and 60% (1). Several techniques for the treatment of OA were introduced over the last years, but most of them did not lead to the anticipated success and were already abandoned [e.g., Marlex® Zipper (2), plastic bags (the Bogota technique) (3), Velcro adhesive sheets (4), sandwich technique (5), modified Barker Vacuum Bag (6)]. Negative pressure wound therapy (NPWT) proved to significantly decrease morbidity and mortality in patients with secondary peritonitis and OA treatment. NPWT activates wound healing, acts as wound fluid drainage, reduces infection and abdominal compartment syndrome (7–9). A “frozen abdomen” and entero-atmospheric fistulas are among possible complications of OA treated with NPWT (10, 11). The retraction of the fascial edges can lead to failure of delayed primarily fascial closure and patients end up with planed hernias (12). Different techniques were established to minimize fascial retraction and facilitate FC like mesh mediated facial traction (13), retention sutured sequential FC (14), or Wittmann patching (15). Dynamical fascial sutures (DFS) reduce fascial retraction and are associated with a high incidence of FC after OA treatment (16, 17).

The duration of OA treatment and the closure technique (component separation, suture technique) influence the outcome of OA treatment (9). Early abdominal closure can lead to reinfection and relaparotomy with subsequent destruction of the fascia, whereas late closure lead to recuts muscle lateralization and complications of abdominal wall reconstruction (1). The prognosis of OA treatment can significantly be improved by NPWT and dynamic closure techniques (18).

Up to know there are some studies in the literature that describe quality of life and incidence of incisional hernias (IH) after OA treatment with NPWT for up to 5 years (19–21). However, long-term outcome studies up to 10 years are rarely found.

In 2017 we published long-term follow-up results of 38 survivors out of 87 patients treated with NPWT and DFS between 2007 and 2012: The median age was 60.9 (25.2–86.1) years, and 17 (44.7%) were females. Twenty-one patients (55.3%) answered the questions about specific long-term complications of OA treatment regarding pain and incisional hernias. Six patients (28.6%) suffered from pain in the previous operating field. Seven (33.3%) patients developed incisional hernias. Three out of seven hernias (42.9%) were treated by surgery.

**Figure 1:**
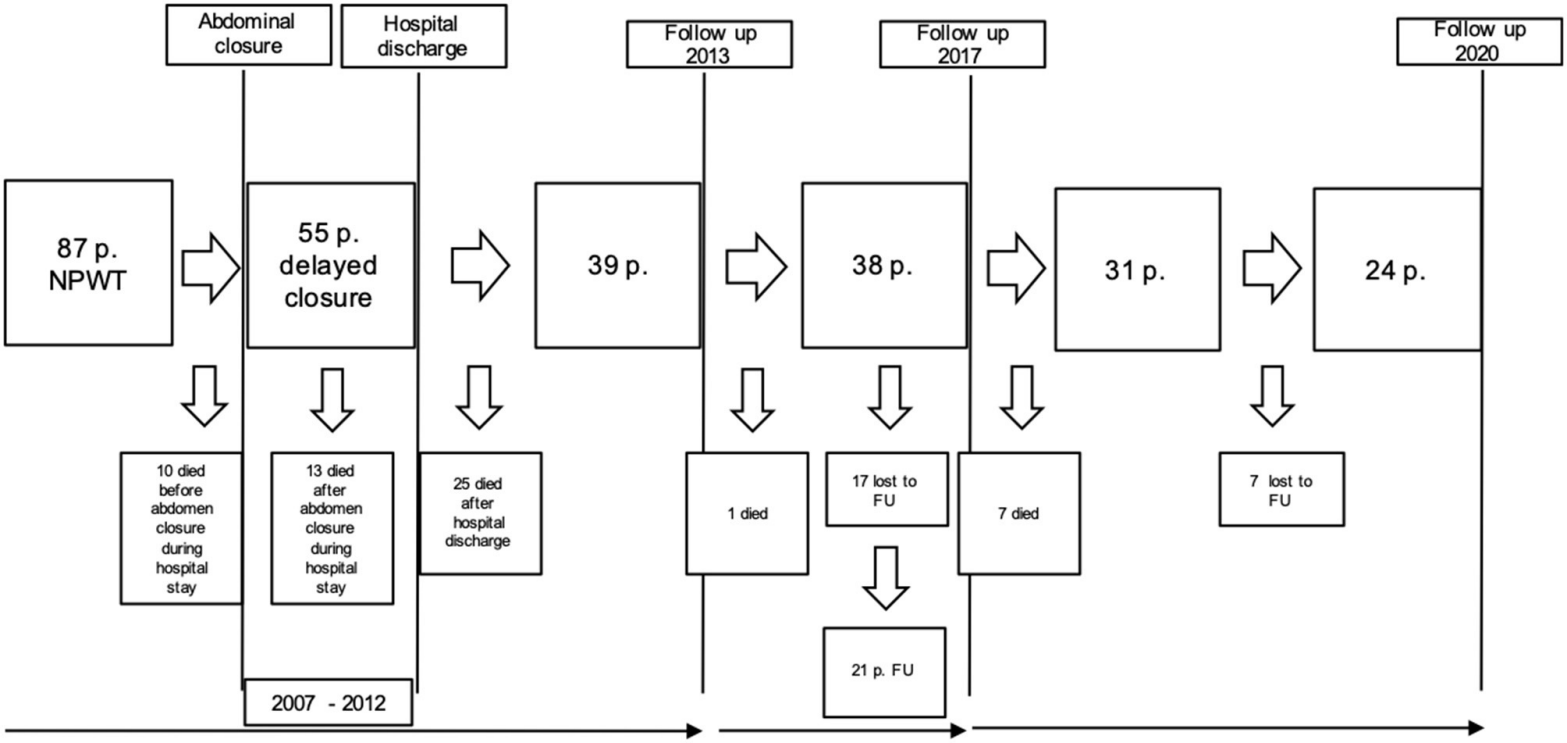
Time line of follow-up (FU): Figure shows number of excluded and included patients over time.

The aim of the present study was to follow-up these patients and to assess their condition 8–13 years after OA treatment with NPWT and DFS (Figure 1). Thirty-eight patients were included in the recent study according to the protocol of our last publication in 2017 (20). After a mean follow-up period of 8–13 years patients were again questioned about long-term complications of OA treatment such as pain, incisional hernia and additional surgical interventions.

Demographic data and further causes of incisional hernias after NPWT therapy were analyzed.

To the best of our knowledge this the longest follow-up study investigating long-term complications of OA treatment with NPWT and DFS.

## Methods

### Study Population

Thirty-eight patients, all survivors from our last study in 2017, were included in the current investigation (20). Inclusion criteria were secondary peritonitis and OA treated with NPWT and DFS between 2007 and 2012 in our hospital. Exclusion criteria were hemorrhage, localized peritonitis, or the ability to perform sufficient source control during the initial procedure in stable patients (17).

Because of the prospective but non-randomized and non-comparative character of the study, the local ethical committee waived responsibility.

### Surgical Technique

The surgical technique of OA treatment with NPWT and DFS has been described in detail in our first publication in 2013 (17).

In brief, after treatment of the source of the initially present secondary peritonitis an intraabdominal vacuum dressing was placed. In order to prevent abdominal muscle lateralization elastic loops were sutured to the fascia in large bites. Patients were admitted to ICU (intensive care unit) and planned reoperations were performed not later than 48 h. After successful NPWT the vacuum dressing and DFS were removed and the abdomen closed with either interrupted or running sutures. Thirty patients out of the included 38 patients in the previous study (78.9%) received FC. In four patients (10.6%) the fascia was not primarily closed and ended up with planned hernias (skin or split- thickness skin graft closure). Fascial closure was performed with running sutures in 26 patients (68.4%), in four patients the fascia was closed using interrupted sutures. In four patients the applied technique was not sufficiently documented (20).

### Questionnaire

**Figure 2:**
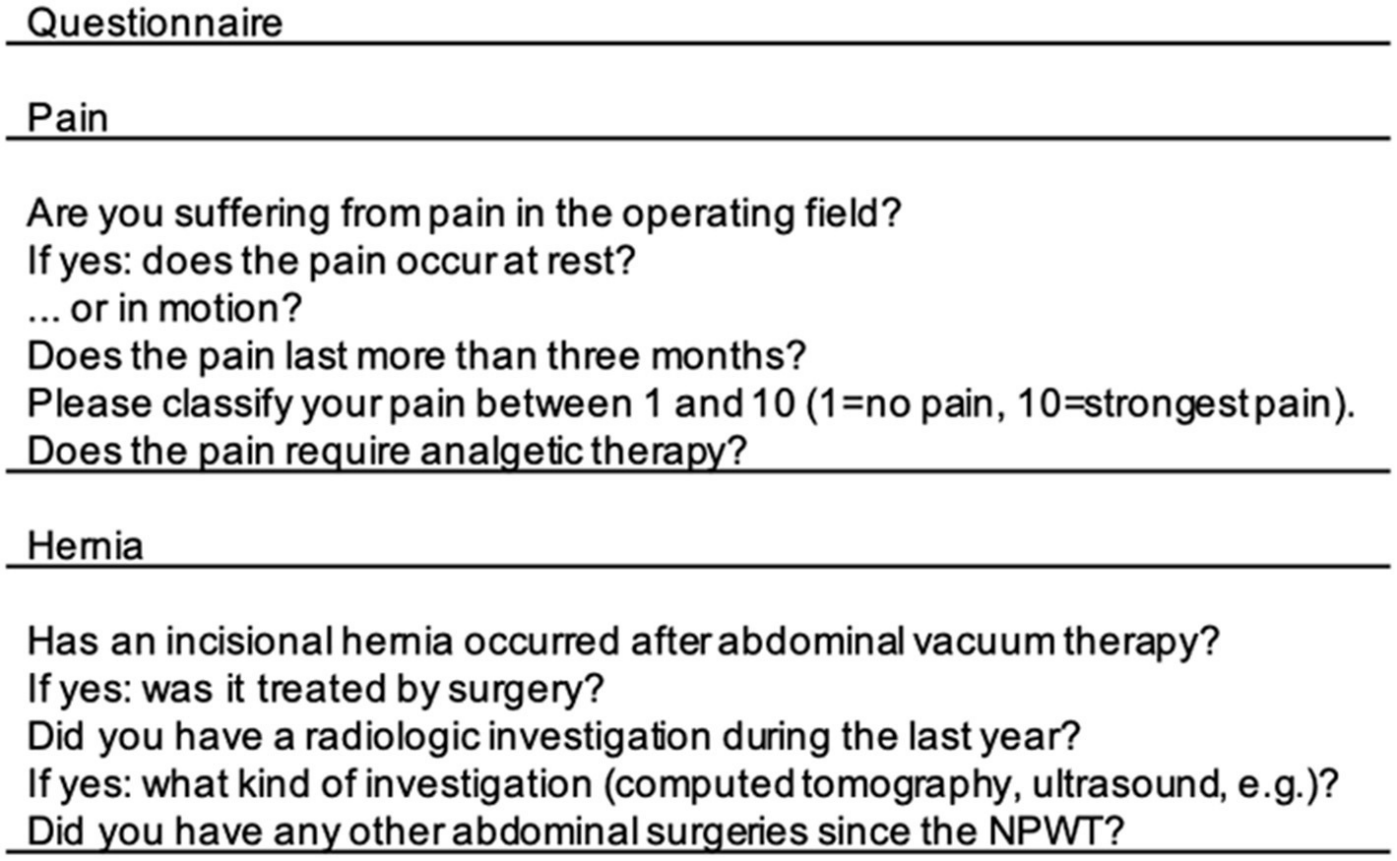
Questionnaire about long-term complications after OA and NPWT. OA, open abdomen; NPWT, negative pressure wound therapy.

A questionnaire regarding long-term complications of OA treatment due to secondary peritonitis between 2007 and 2012 were sent to the included patients. Patients were asked about pain in the operating field differentiating between acute and chronic pain and the necessity of analgesic treatment. The questionnaire further targeted potential IH after DFS, hernia diagnostics and treatment or other surgeries in the previous operating field. In case of not returned questionnaire within 4 weeks the patients were contacted for an oral interview. Questions are listed in Figure 2. Patients we were not able to get in touch with were lost to follow-up.

### Data Collection

We collected the following data: patients' demographics, MPI, ASA classification (American Society of Anesthesiologists), Simplified Acute Physiology Score (SAPS), number of NPWT changes, duration of NPWT, and duration of ICU stay in days.

The results were analyzed quantitatively and presented as median and range, unless otherwise stated.

Statistical testing was carried out using the Kolmogorov–Smirnov Test for Gaussian distribution. *t*-test was used when comparing two groups and when data were normally distributed, Mann–Whitney test when data were not normally distributed. Contingency was tested with Chi-square (>2 variables) and Fisher's tests (two variables, low n). *p*-values of <0.05 were considered to indicate statistical significance.

## Results

### Demographic Data

Since our study in 2017 seven more patients had deceased (four women, three men) with a median age of 78 years (69–96 years). The causes of death were not documented, but the main reason might have been natural death due to the patients' high age.

Thirty-one patients were included in the current study, 15 patients were females (48.4%) and 16 were males (51.6%) with a median age of 69 years (33–90 years). The overall mortality rate after the follow-up duration starting in 2007 with 87 included patients was 64.4%. The median MPI as index for intraperitoneal peritonitis at the timepoint of the initial surgery was 14 (5–26), the median ASA as index for co-morbidities was 2 (1–4) and the SAPS as index for physiological health was 8 (0–28) in the current study population (Table 1).

**Table 1:** Patients demographic data.

Population	31
Male sex	16 (51.6%)
Female sex	15 (48.4%)
Age	69 (33–90)
Source of Infection	
Upper GI	10 (32.2%)
Lower GI	19 (61.3%)
Pancreas	2 (6.5%)
MPI	14 (5–26)
SAPS	8 (0–28)
ASA	2 (1–4)
Reoperations	3 (1–16)
NPWT duration	6.5 (3–62)
ICU stay	13 (3–74)

*GI, Gastrointestinal tract; MPI, Mannheimer Peritonitis score; SAPS, Simplified Acute Physiology Score; ASA, American Society of Anesthesiologists; NPWT, Negative Pressure Wound Therapy; ICU, Intensive care unit*.

**Figure 3:**
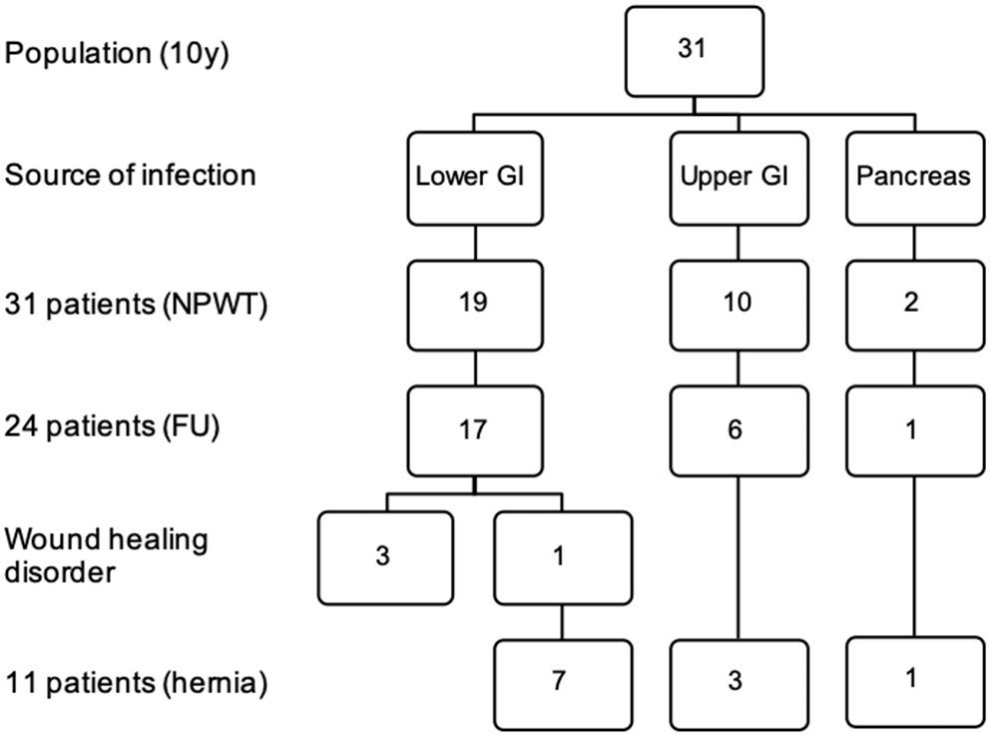
Source of infection, wound healing disorder and incisional hernia in a 10 years follow up. FU, follow-up; NPWT, negative pressure wound therapy; GI, gastrointestinal tract.

The source of infection at the initial surgery was the upper GI (gastrointestinal) tract (stomach, duodenum, small bowel) in 10 patients (32.3%), the lower GI tract (colon, rectum) in 19 patients (61.3%) and pancreas in two cases (6.5%) (Figure 3).

The follow-up period was 8–13 years.

### NPWT and Delayed Closure

The median NPWT duration was 6.5 days (3–62) with a median of 3 (1–16) reoperations (Table 1). In 27 patients (87.1%) FC could be performed, in four patients (12.9%) FC was not possible: in two patients the skin only could be closed, in one case split-thickness skin grafts were used for abdominal closure. These three patients had planned hernias after discharge from hospital. One patient received plastic abdominal wall reconstruction.

**Figure 4:**
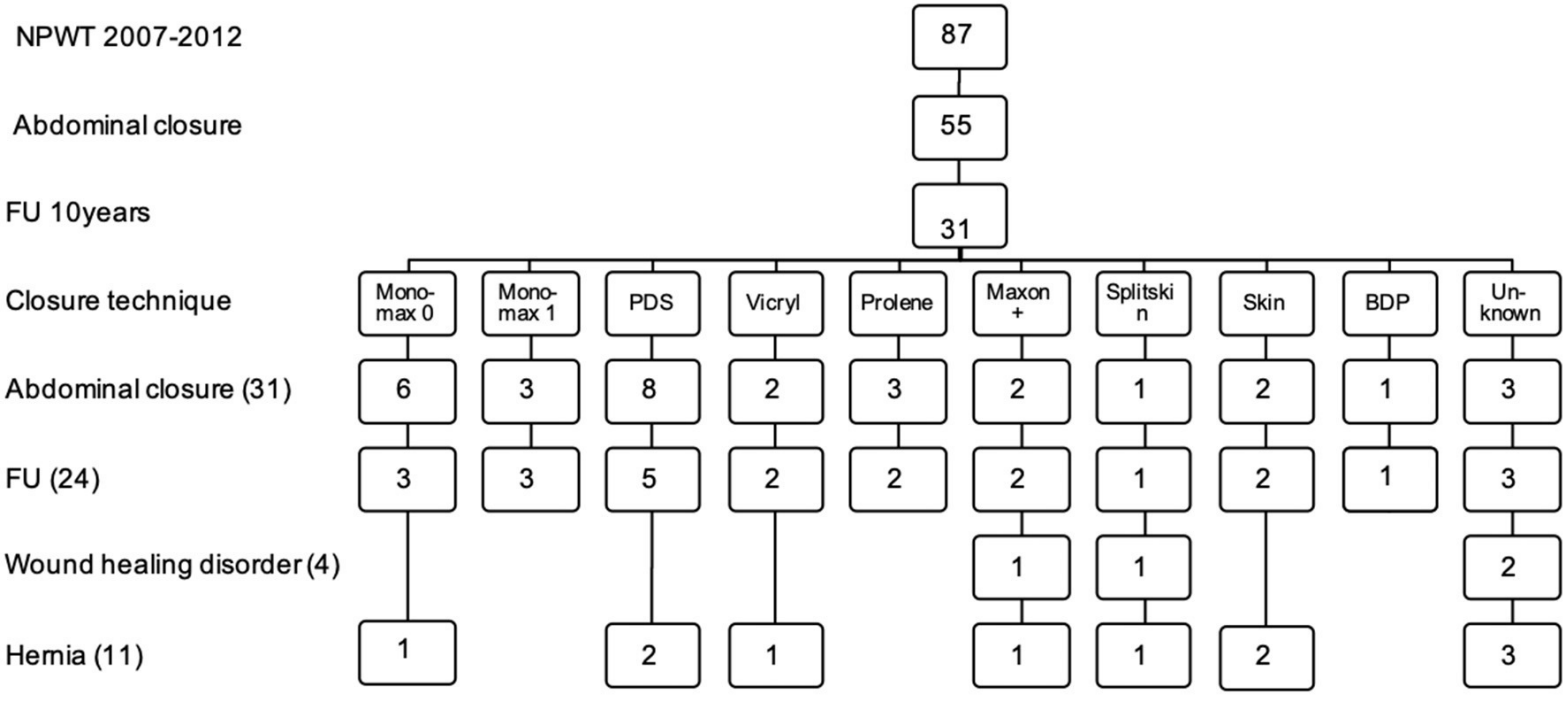
Techinque of delayed abdominal closure, wound healing disorder and incisional hernia in a 10 years follow up. FU, follow-up; NPWT, negative pressure wound therapy.

Fascial closure with running sutures was performed in 22 patients: Six with Monomax® 0 (Braun, Melsungen, Germany), three with Monomax® 1 (Braun, Melsungen, Germany), eight with PDS® (Ethicon, Norderstedt, Germany), two with MaxonPlus® (Braun, Melsungen, Germany), and three with Prolene® (Ethicon, Norderstedt, Germany). In two patients the fascia was closed with interrupted sutures using Vicryl® (Ethicon, Norderstedt, Germany). In three patients the applied technique was not sufficiently documented (Figure 4). The median stay at the ICU was 13 (3–74) days.

### Questionnaire

Among the 31 included patients 24 (77.4%) answered the questionnaire (Figure 2).

Three patients (12.5%) were still suffering from pain in the operating field. All three patients were having pain at rest but not during exercise typical of the respective patient's age and for more than 3 months (chronic pain). None of the patients required analgesic treatment. The mean VAS score was 3 (3–4). Twenty-one patients (87.5%) did not feel any pain in the original operating field (Table 2).

**Table 2:** Results of the questionnaire answered by 24 patients.

Pain	3	12.5%
Pain at rest	3	
Pain in motion	0	
Chronic pain (>3 months)	3	
VAS (1–10)	3.3	3–4
Analgetic therapy required	0	
Incisional hernia	11	45.8%
Clinically detected	5	45.5%
Radiologically detected	6	54.5%
Hernia repair	5	45.5%
Recurrence	1	
Other abdominal surgeries after NPWT	6	25%

Among the 24 patients, 11 patients (45.8%) have developed an IH, whereas three patients suffered from planned IH after skin closure only or split thickness skin grafts. Six hernias (54.5%) were diagnosed radiologically (computed tomography, magnetic resonance imaging, ultrasound), the other five hernias (45.5%) were diagnosed by clinical examination. Five patients (45.5%) with hernias underwent surgical hernia repair, with one recurrence hernia among the operated patients. None of these patients claimed pain in the operating field (Table 3).

**Table 3:** Answers regarding incisional hernias, with and without repair and pain.

	**Pain (3)**	**No pain (21)**
Hernia (11)	2 (18.2%)	9 (81.8%)
No hernia (13)	1 (7.7%)	12 (92.3%)
Hernia (w. repair) (5)	0	5 (100%)
Hernia (wo. repair) (6)	2 (33.3%)	4 (66.7%)

**Figure 5:**
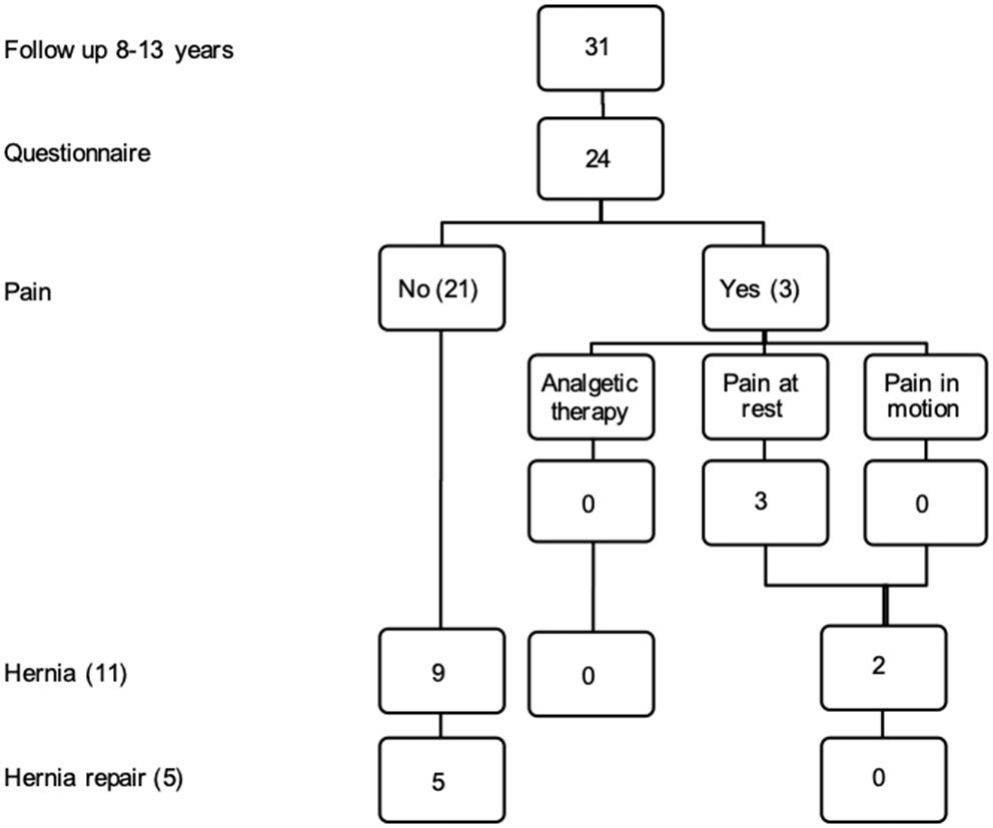
Twenty-four patients were available for follow up. Flow chart regarding pain incisional hernia and hernia repair.

Among the 11 IH patients nine (81.8%) were found to have an asymptomatic hernia and two (18.2%) symptomatic hernias. Two out of three patients with pain in the operating field (66.6%) still had an IH and did not have hernia repair (Table 3, Figure 5).

Six patients (25%) underwent additional abdominal surgery after NPWT and fascial or skin closure: one prostatectomy, two stoma surgeries, one scar correction, one cholecystectomy, one reason for surgery was not reported.

The reason for loss to follow-up were changes in address and/or phone number, or personal reason not to answer our questionnaire.

### Incisional Hernia

We compared the technique of fascial closure, the source of infection, delayed wound healing in patients that developed an IH after NPWT with DFS.

Among the 24 patients, 11 patients (45.8%) were found to have an IH. Four patients (36.4%) received FC with running sutures: one with Monomax® 0 (9%), two with PDS® (18.2%) and one with MaxonPlus® (9%). In one patient (9%) the fascia was closed with interrupted sutures (Vicryl®). In three patients the technique of FC was not documented (27.3%). Three patients did not receive FC after NPWT therapy but skin closure only or split thickness grafts (27.3%) (Figure 4).

One patient that was found to have an IH after NPWT has died since our last publication and was excluded in the present study.

The source of infection of the secondary peritonitis among the IH patients was documented: In three out of ten patients (30%) with the upper GI tract (stomach, duodenum, small bowel), in seven among 19 patients (36.8%) with the lower GI (colon, rectum) and in one out of two patients (50%) with the pancreas as source of infection an IH was diagnosed (Figure 3). No significant relation could be shown between source of peritonitis and the development of IH.

Four patients suffered delayed wound healing after NPWT, one of these patients did not receive FC but skin closure and one patient with delayed wound healing developed an IH. All four patients had the lower GI as source of infection (Figure 3), but not all of these were found to have an IH.

Delayed abdominal closure in patients with IH was achieved after 10 (3–62) days with 4 (1–16) reoperations. Patients without IH received delayed closure after 6 (3–38) days with 3 (1–10) reoperations (Table 4). No significant differences were detected between treatment days, reoperations and the development of IH (*p* = 0.302 and *p* = 0.238). The mean MPI in patients that developed an IH [11 (5–26)] was lower compared to the patients without IH [15(9–25)] but failed to be significant (*p* = 0.107). The mean SAPS and ASA score was lower in patients that have developed an incisional hernia [7 (1–15) vs. 11 (4–28), 1 (1–3) vs. 2 (1–4)] (*p* = 0.66, *p* = 0.92). The mean stay at ICU was lower in patients with IH [13 (3–74) vs. 15 (11–39)] (*p* = 0.1) (Table 4). The mean BMI was higher in patients with IH [28 (16–44)] compared to patients without IH [27 (18–35)] but without significant differences (*p* = 0.564).

**Table 4:** Possible risk factors for the development of incisional hernia (IH) after NPWT.

	**IH**	**No IH**
NPWT (days)	10 (3–62)	6 (3–38)
Reoperations	4 (1–16)	3 (1–10)
ICU (days)	13 (3–74)	15 (11–39)
MPI	11 (5–26)	15 (9–25)
SAPS	7 (1–15)	11 (4–28)
ASA	1 (1–3)	2 (1–4)
BMI	28 (16–44)	27 (18–35)

*MPI, Mannheimer Peritonitis score; SAPS, Simplified Acute Physiology Score; ASA, American Society of Anesthesiologists; NPWT, Negative Pressure Wound Therapy; ICU, Intensive care unit; BMI, Body Mass Index*.

## Discussion

After a follow-up period of 8–13 years 31 out of 87 primarily included patients (35.6%) were still alive. Seven more patients died since our last study in 2017. The overall mortality rate over the whole follow-up period is 64.4%, whereas 26.4% of the patients already died during the hospital stay. We already described a high early mortality rate and detected significantly higher MPI, ASA and SAPS scores in the patient population that did not survive until the first follow-up. After these first critical months OA and NPWT is accompanied by a satisfying survival rate (20). Currently there are no data in the literature about survival rates over a follow-up period of 10 years.

In the current study 24 among 31 patients (77.4%) were available for follow-up compared to 21 out of 36 (55.3%) in our study in 2017. In 11 patients an incisional hernia was diagnosed (45.8%). In our previous study a lower IH rate with seven hernias among 21 patients (33.3%) were detected. Additionally, two of the IH patients died since 2017 and were excluded in the present study. The higher rate of IH can be explained by two recently diagnosed hernias and four patients with hernias that were not available for follow-up in the previous study.

The hernia rate of 45.8% after a period of 8–13 years is still lower than described by Petersson et al. who found an IH rate of 62% in a 5-years follow-up after OA treatment with vacuum assisted wound closure and mesh mediated fascial traction. The mean time of hernia diagnosis was 11 months, either by clinical investigation (36%) or CT scan or laparotomy in further 30%. The earlier development of IH can be explained by a more extensive injury to the fascia caused by suturing the mesh to the fascial edges (19).

Brandl et al. (12) found an IH rate of 35% at a median follow-up time of 26 (12–81) months using NPWT for OA treatment without DFS or other devices to reduce fascial lateralization. The Kaplan-Meier analysis estimated a hernia rate of 66% after five years, which is higher compared to our study with 45.8% after 7–13 years. They described the best results with running sutures and a slow absorbable material for DFS and the highest IH rate with interrupted sutures and absorbable material (Vicryl®). They discuss a certain selection bias with a higher rate of Vicryl® use in patients with a higher extent of fascial contamination and injury, and higher degree of tension at closure. In our study DFS were applied to reduce fascial lateralization to lower the consecutive tension at the time of FC. The higher fascial tension and the use of different closure techniques can explain the differences in IH rates described by Brandl et al. (12). A follow-up study with recent data might be of interest.

The recommended closure technique for midline closure described by Israelsson et al. is a running suture in small bite technique with 5:1 or 6:1 suture-to-wound length (22–24). We changed closure technique according to the recommendations from interrupted sutures with multifilament, resorbable and non-resorbable material *(Vicryl*^®^*, Ethibond*^®^*)* to large bite and small bite technique with monofilament, non-resorbable *(Prolene*^®^*)* and late resorbable sutures *(PDS*^®^*, MaxonPlus*^®^*, MonoMax*^®^*)*. This change caused a heterogeneity in closure technique in our study, thus recommendations for FC according to our study results are hardly possible.

Petersson et al. performed FC with PDS® achieving a suture to wound ratio of at least 4:1 after mesh mediated fascial traction and found higher IH rates compared to our study (19). This might well be due to the above mentioned more extensive fascial injury rather than to the actual suture technique.

Jakob et al. described a new technique for abdominal closure after OA treatment. After an initial period with NPWT they implanted an intraperitoneal onlay mesh (VAC-IPOM). The fascia was partially or completely closed with running sutures using PDS® and NPWT was applied to the mesh. NPWT was kept until an adequate formation of granulation tissue was achieved. They compared their new technique with vacuum-assisted wound closure and mesh-mediated fascial traction (VAWCM) with direct fascial closure using absorbable loops. They described less re-operations and reduced hospital and ICU stay after VAC-IPOM therapy. Complete fascial closure using VAC-IPOM was achieved in only 26% compared to 74.2% using VAWCM. 25.8% of patients with VAWCM were left with a planned hernia. They described a significantly longer hernia-free survival using their new technique caused by a possible sufficient stabilization using the IPOM mesh without direct fascial closure and a subsequent reduction of extensive fascial tension. This reduced fascial tension and the possible fascial injury as well as the use of resorbable material in the VAWCM group can explain the difference in hernia incidence (21). A comparison to our study is hardly possible, because the authors do not offer an IH rate at a given time point.

Willms et al. recently published a multi-center multivariable analysis of data from the Open Abdomen Route of the European Hernia Society. They found a significant improvement of prognosis of OA and a positive correlation of fascial closure with NPWT and dynamic closure techniques. A high intraabdominal contamination and long treatment before facial closure was found to be negative correlated with fascial closure (18).

In our previous study we found significantly fewer NPWT treatment days and reoperations in patients without IH (20). These findings indicate a possible negative influence of OA and NPWT on hernia development. In the recent study patients with IH achieved delayed abdominal closure after 10 (3–62 days) and 4 (1–16) reoperations. Patients without IH were again found to have fewer NPWT treatment days [6 (3–38)] and reoperations [3 (1–10)]. Nevertheless, after a mean of 10 years no significant differences could be detected between treatment days, reoperations and the development of hernias, although we still see a clear trend. Interestingly and in contrast to the results regarding reoperations and treatment days, we detected a lower MPI, lower ASA and SAPS scores and fewer days at ICU in patients that developed an incisional hernia over a time period of 8–13 years. Hence, patients without IH had a higher MPI but a shorter treatment period. These findings might support a trend to fewer reoperations and treatment days despite distinct secondary peritonitis at the time of acute primary surgery. The BMI at time of primary surgery was lower in patients that did not develop an incisional hernia without significant differences. Bjarnason also did not find a significant association between known risk factors of IH (e.g., obesity) and the development of IH one year after OA treatment but discussed a possible type II statistical error (25). Interestingly, all four patients with wound healing disorder had the lower GI as source of infection, but not all of these patients were found to have an IH in the FU. Wound healing disorder is a well-known risk factor for IH, but might lose its importance in the development of IH in a long-term FU.

Three out of 24 patients were still suffering from pain in the original operating field, one patient had three further abdominal surgeries, the other two patients achieved abdominal closure after NPWT with split skin grafts and planned hernias. None of the patients required ongoing pain medication. Five patients with IH had undergone incisional hernia repair. In contrast to our previous study none of the patients complained of pain in the operating field. Among the six patients that did not have hernia repair four do not claim pain in the operating field. We reported of six patients with pain in our previous study with a possible relation of pain and IH. Bjarnason et al. reported similar relations between pain and IH after NPWT and mesh-mediated fascial traction after 1 year (25). Petersson at al found 59% of patients complaining of different symptoms at the abdominal wall without relation to IH in the same study population after 5 years (19). Only 14% reported pain in the original operating field. These findings and our own results suggest a possible reduction of pain over time. Abdominal symptoms might be caused by possible consequences of secondary peritonitis and OA with NPWT (e.g., adhesions).

Incisional hernias remain a serious problem after OA and NPWT. Our long-term follow-up study over up to 13 years underlines the relevance of incisional hernias as a main long-term complication after OA treatment. Nevertheless, the use of dynamic fascial sutures and negative pressure wound therapy lead to high rates of success in delayed fascial closure, fewer hernias and a low incidence of pain compared to other techniques. To the best of our knowledge our study reviews the longest follow-up period of 8–13 years. Due to a seriously ill and elderly patient population we found a high drop-out rate. Recommendations for FC technique in this special case of partly injured fascia and lateralization of the rectus muscle are limited by the heterogeneity of FC in our study.

In conclusion, the incidence of incisional hernias after OA treatment increases over time with 45.9% after a mean follow up of 10 years. In contrast, the number of patients with pain in the original operating field and the use of analgesic treatment decreases over time. Further research is needed to investigate techniques for fascial closure after NPWT and an eventual positive effect with standardized methods has to be hypothesized.

## Data Availability Statement

The original contributions presented in the study are included in the article/supplementary material, further inquiries can be directed to the corresponding author/s.

## Ethics Statement

Ethical review and approval was not required for the study on human participants in accordance with the local legislation and institutional requirements. Written informed consent for participation was not required for this study in accordance with the national legislation and the institutional requirements.

## Author Contributions

AH tasks were literature research, send and analyse questionnaires, analyses of all data, and manuscript writing. CM's tasks were writing and proof reading. KG and RF were responsible for support in text writing and analyses, and proof reading. All authors contributed to the article and approved the submitted version.

## Conflict of Interest

The authors declare that the research was conducted in the absence of any commercial or financial relationships that could be construed as a potential conflict of interest.

## Abbreviations

OA: Open Abdomen
NPWT: Negative Pressure Wound Therapy
DFS: Dynamic Fascial Sutures
FC: Fascial Closure
IH: Incisional Hernia
MPI: Mannheimer Peritonitis Index
SAPS: Simplified Acute Physiology Score
ASA: American Society of Anesthesiologists
ICU: Intensive care unit
VAS: Visual Analog Scale.
